# Genome-Wide Association Studies and Runs of Homozygosity Reveals Genetic Markers Associated with Reproductive Performance in Korean Duroc, Landrace, and Yorkshire Breeds

**DOI:** 10.3390/genes15111422

**Published:** 2024-10-31

**Authors:** Kefala Taye Mekonnen, Dong-Hui Lee, Young-Gyu Cho, Ah-Yeong Son, Kang-Seok Seo

**Affiliations:** 1 Department of Animal Science and Technology, Sunchon National University, Suncheon 57922, Republic of Korea; kefala.taye@arsiun.edu.et (K.T.M.);; 2 Department of Animal Science, College of Agriculture and Environmental Science, Arsi University, Asella 193, Ethiopia

**Keywords:** breed, candidate genes, genome, GWAS, Korea, NBA, run of homozygosity, TNB

## Abstract

Background: Reproductive performance is critical in the pig industry, and improved sow performance could lead to increased economic benefits. GWAS and ROH analyses based on SNP array data were conducted to identify the breed-specific genetic architecture underlying the variation in NBA and TNB. Methods: A total of 7488 breeding pigs with phenotypic data from 1586 Duroc, 2256 Landrace, and 3646 Yorkshire breeds, along with 76,756 SNP markers from Korean grand-grand-parent (GGP) breeding farms, were used. Results: In the Duroc breeds, SNPs on *SSC* 9 and 17 were found to be associated with the *SIDT2* and *TGM2* genes, respectively. In the Landrace breed, *PPP1R9A*, *LMTK2*, and *GTF2H3* on *SSCs* 9, 3, and 14, respectively, were associated with both TNB and NBA. With the Yorkshire breed genome, *GRID1*, *DLGAP2*, *ZZEF1*, *PARG*, *RNF17*, and *NDUFAF5* in SSCs 14, 15, 12, 14, 11, and 17, respectively, were associated with NBA and TNB traits. These genes have distinct functions, ranging from synaptic transmission and cytoskeletal organization to DNA repair and cellular energy production. In the Duroc breed, six genes identified in the ROH islands were associated with various biological pathways, molecular functions, and cellular components. *NT5DC1* was associated with metaphyseal chondrodysplasia, *CRTAC1* with ion binding, *CFAP43* with spermatogenic failure, *CASC3* with intracellular mRNA localization, *ERC2* with cellular component organization, and *FOCAD* with Focadhesin. In the Landrace and Yorkshire breeds, *PDE6D* was associated with GTPase inhibitor activity. Conclusions: Through GWAS and ROH analyses, we identified breed-specific SNP markers associated with NBA and TNB in three breed genotypes, providing insights for improving reproductive performance efficiency and contributing to future breeding strategies.

## Simple Summary

Genome-wide association studies (GWAS) and run of homozygosity (ROH) analyses based on single nucleotide polymorphism (SNP) data were powerful tools for uncovering genetic factors associated with reproductive performance efficiency in various livestock breeds. The effectiveness of these studies may differ based on breed-specific characteristics, population genetics, and targeted production traits. In the present study, comparative analyses of the genetic profiles of pigs that exhibit diverse reproductive performance were conducted. This empowered us to identify genomic regions associated with the number of piglets born alive (NBA) and the total number of piglets born (TNB). The present finding greatly enhance its contributions to revisit breeding strategies in pigs by identifying significant genetic markers associated with reproductive performance traits.

## 1. Introduction

Improving pig reproductive capabilities has been a consistently fascinating subject in the swine breeding and farming industry [1]. Molecular breeding, a method that involves the identification of single nucleotide polymorphism (SNPs) and prospective genes linked to reproduction, has been demonstrated as an effective approach to enhancing reproductive performance [2]. Reproductive traits, such as total number born (TNB) and number born alive (NBA), are crucial factors used in pig breeding programs to assess sow productivity [3]. So far, the use of classical breeding methods with the best linear unbiased prediction has been effective in enhancing maternal reproductive attributes [4]. However, factors such as low heritability, minor genes, maternal effects, and environmental factors primarily influence the intricate genetic structure of reproductive traits [5]. Consequently, unraveling the genetic architecture of reproductive performance traits poses significant challenges. GWAS and ROH analyses are the major genomic tools applicable to identify genes related to reproductive performance in livestock breeds in which pigs are not exceptional. However, it varies depending on the breed, population, and production traits. Moreover, there is a belief that GWAS signals have been replicated across populations from various regions [6], which has been substantiated by several reported studies [7,8,9].

Reproductive performance is a crucial production trait in the swine business [10]. TNB and NBA are essential markers for assessing reproductive performance, which in turn has a considerable impact on the overall performance of the pig industry and sow evaluation programs [3]. Improving the reproductive performance of sows can result in higher economic benefits for the swine industry [11]. To date, traditional breeding strategies that use the best linear unbiased prediction have been effective in enhancing maternal reproductive attributes [4]. However, because of their low heritability, minor genes, maternal effects, and environmental influences, their genetic architecture is substantially more complex [5], making it difficult to decipher the genetic architecture of reproductive traits [12]. Hence, typical breeding strategies are ineffective for rapidly improving these features [11,13]. With the advancement of molecular breeding technology, marker-assisted selection (MAS) and genomic selection (GS) have emerged as viable strategies for increasing swine breeding efficiency [14].

ROH are cutting-edge methods for analyzing inbreeding in livestock populations [15]. ROH are contiguous regions on chromosomes in which an individual inherits identical haplotypes from both parents [16,17,18,19,20]. These long, unbroken, homozygous areas in the genome are likely to have evolved from a common ancestor [21]. They are useful for predicting inbreeding rates and analyzing genetic linkages across the entire genome of livestock species [22,23,24]. When both parents pass down substantial chromosomal segment portions, the offspring’s genome contains homozygous segments. Factors such as inbreeding, genetic drift, consanguineous mating, population bottlenecks, and natural and artificial selection influence these segments among both inbred and non-inbred populations [17,25,26,27,28,29,30,31]. Recent advances in high-throughput genomic analysis tools, including next-generation sequencing and genotype-based microarrays (SNP arrays), have made it easier to identify these homozygous portions with higher resolution [17,32].

Therefore, identifying breed-specific genes related to reproductive performance is crucial. This could be achieved by conducting GWAS and analyzing ROH in three pig populations with distinct genetic backgrounds based on SNP array data. Our study had two main objectives: first, to conduct GWAS and identify genes associated with TNB and NBA in the Korean Duroc, Landrace, and Yorkshire pig breeds and, second, to explore genes and their functions in genomic regions associated with ROH islands and pathways contributing to sow reproductive performance in these breeds. Our GWAS and ROH analyses revealed valuable candidate genes that influence pig reproductive performance traits directly or indirectly, especially TNB and NBA. We inform breeders that this study contributes to the improvement of productivity and the development of effective breeding programs, thereby enhancing overall productivity.

## 2. Materials and Methods

### 2.1. Animals and Phenotype

In this study, we performed GWAS on the key economic traits of reproductive performance, using genomic markers (single nucleotide polymorphisms, SNP) and phenotypic data gathered from Korean Duroc, Landrace, and Yorkshire breeds at GGP domestic breeding farms. The analysis included information on reproductive performance, specifically NBA and TNB. The reproductive performance data from a total of 7488 breeding pigs, comprising 1586 Duroc, 2256 Landrace, and 3646 Yorkshire breeds, was collected. Additionally, a dataset containing 76,756 SNP markers was utilized. Table 1 presents the descriptive statistics of NBA and TNB for the Korean Duroc, Landrace, and Yorkshire breeds. 

### 2.2. Genotyping and Quality Control

Genotyping was performed using SNP markers, and blood samples were used for DNA collection. The porcine 70K SNP chips (Illumina, San Diego, CA, USA) were used. Processed genotypic data were obtained following the internal guidelines specified by the data provider. In total, 7488 breeding pigs (1586 Duroc, 2256 Landrace, and 3646 Yorkshire pigs) and 76,756 SNP markers were included in the collected basic data. For quality control, PLINK software (v1.90) was used to keep SNPs with MAF > 0.05, mind 0.1, SNP call rate > 95%, individual call rate > 5%, and HWE > 1 × 10^−6^ [33]. A total of 57,775 SNPs and 7413 animals were retained for further GWAS analysis.

### 2.3. Phenotypic Correlation Analysis

The reproductive performance traits of the Duroc, Landrace, and Yorkshire pig breeds were compared using Spearman’s correlation analysis to ensure the robustness of the correlation results. R version 4.3.3 (2024-02-29 ucrt) “Angel Food Cake” was used for analysis and visualization. Phenotypic correlation analysis and visualization were performed using the "*ggscatmat*" function from the “*GGally*” package. In addition to the default function, additional formatting and customization options were applied to enhance visualization. Notably, the results of the correlation analysis were reported in two significant digits, ensuring clarity and precision in the presentation of the findings.

### 2.4. Principal Component Analysis (PCA)

The three distinct breed populations examined in this study had different genetic backgrounds, and we used GCTA software v1.94.1 developed by Yang, Lee [34] to conduct PCA to differentiate the population stratification. We performed principal components to assess genetic differentiation between the three breeds. Accordingly, we calculated the proportion of variance accounted for by the first principal component by dividing the variance explained by the total variance. Based on the variance explained by the PCA results (principal component 1 vs. principal component 2 and principal component 1 vs. principal component 3), we determined the number of principal components to include as covariates. Therefore, we included the first two principal components (principal component 1 and principal component 2) as covariates in the GWA studies.

### 2.5. Genome-Wide Association Study

We examined the association between reproductive performance traits and reliable SNP markers using the linear regression module of PLINK1.9 software v1.94.1 [33]. As discussed in Section 2.2, the SNP datasets for each breed yielded 7941, 8409, and 8394 SNPs for the Duroc, Landrace, and Yorkshire breeds, respectively. We used the first two PCs as covariates in the GWAS. We used the Bonferroni correction to identify significant SNPs at a 0.05 significance level. The *p*-values for the 5% genome-wide significance thresholds in the three populations were calculated as 0.05/SNPs. We used a Bonferroni correction to adjust the *p*-values for multiple tests. The genome-wide significance threshold was set at *p* < 0.05 and divided by the number of SNPs for each breed: *p* < 6.29 × 10^−6^ for the Duroc breed, *p* < 5.94 × 10^−6^ for the Landrace breed, and *p* < 5.95 × 10^−6^ for the Yorkshire breed. Also, chromosome-wide significance thresholds were set at *p* < 6.29 × 10^−7^ for the Duroc breed, *p* < 5.94 × 10^−7^ for the Landrace breed, and *p* < 5.95 × 10^−7^ for the Yorkshire breed. These levels have suggestive significance. Manhattan and quantile–quantile (Q–Q) plots were generated using the R package “*qqman*” to visualize the significance of the *p*-values of the GWAS for candidate genes and to detect the population stratification [35], respectively.

### 2.6. Identification and Annotation of Candidate Genes from GWAS

Pertinent information on potential genes was accessed from the Ensembl Sscrofa11.1 database (www.ensembl.org accessed on 25 April 2024) at http://www.ensembl.org. The Ensembl database provides comprehensive genomic information on various species, including pigs (*Sscrofa*). The candidate genes underwent gene ontology (GO) annotation analysis (http://geneontology.org accessed on 25 April 2024). GO annotation involves assigning functional annotations to genes based on their biological processes, molecular functions, and cellular components. To validate gene function, we conducted a comprehensive search of the additional literature from PubMed to obtain more detailed information on gene function.

### 2.7. Gene Exploration of Shared ROH Regions

We selected homozygous segments shared by >70% of the samples as potential indications of ROH islands across the genome. We used the ENSEMBL database, based on the pig reference genome version 11.1, to annotate functional genes within the ROH islands. This approach allowed for the identification and analysis of the most common genomic regions associated with ROHs. To identify significant candidate genes, GO and KEGG pathways with a significance level of *p* ≤ 0.05 were used [36,37]. To gain insight into the functional implications of the identified ROH islands, we utilized a list of genes from the ROH islands and the *Sus scrofa* annotation file as the reference background.

## 3. Results

### 3.1. Phenotype Correlation of Reproductive Traits 

**Figure 1:**
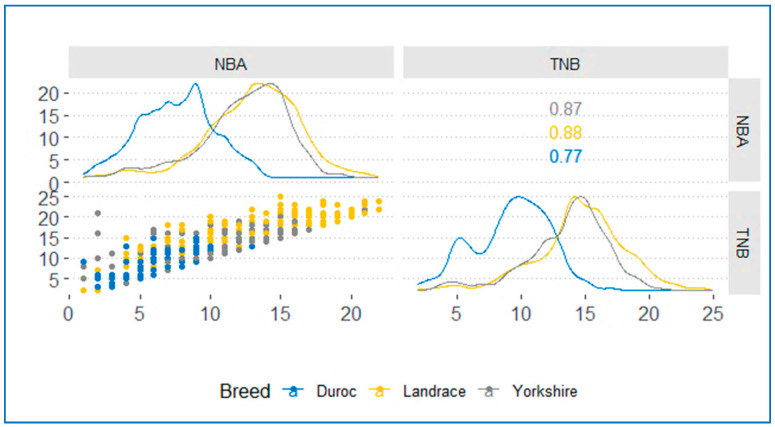
Spearman’s correlation coefficient between NBA and TNB traits in the three breeds. The three distinct colors represent traits from three different breeds.

Figure 1 displays the phenotypic correlation for NBA and TNB traits in the three pig populations (Duroc, Landrace, and Yorkshire). For all breeds, Spearman’s correlation coefficient among the traits showed a strong positive correlation, indicating that they were highly correlated. The Landrace breed had the relatively highest correlation (0.88) for the trait under study, followed by Yorkshire (0.86) and Duroc (0.77). This observation highlights the strong correlation between NBA and TNB, reinforcing our understanding that maximizing improvements in NBA can lead to an increase in TNB. Furthermore, it is worth noting that genes influencing NBA and TNB in all three breeds may exhibit similar effects. Therefore, this potential interconnectedness suggests that a gene that affects one trait may also affect other traits and may have a shared genetic factor.

### 3.2. Principal Component Analysis (PCA) and Population Structure 

**Figure 2:**
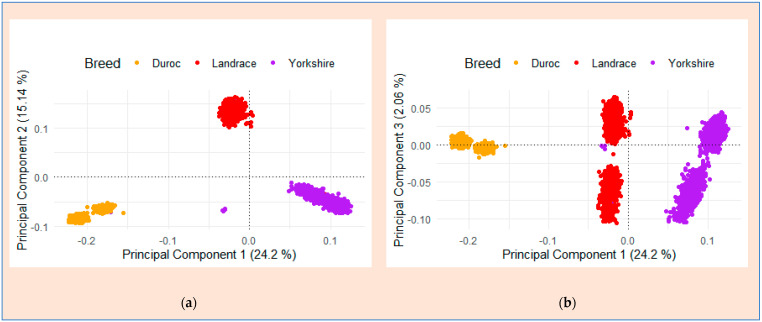
Visualization of the dataset of the first three principal components of the PCA plot illustrating genotype clustering in the three Korean commercial pig breeds using SNPs. The PCA plot displayed individuals clustered based on their genetic similarities: in (**a**), the variance is explained by principal component 1 (24.2%) and principal component 2 (15.14%), and in (**b**), the variance is explained by principal component 1 (24.2%) and principal component 3 (2.06%).

Population stratification is a key consideration in genome-wide association studies because it might induce consistent ancestry disparities, conceivably resulting in spurious associations [38]. To identify the population structure of the three breeds from Korean GGP breeding farms, we performed PCA using SNP data. Using a PCA plot, we differentiated population stratification based on the genetic similarities among three commercial pig breeds from Korean GGP breeding farms (Duroc, Landrace, and Yorkshire) using principal component analysis (PCA) of the SNP data (Figure 2a,b). PCA revealed clear genetic clustering, with the first two principal components (PCs) delineating three distinct clusters corresponding to each breed (Figure 2a), explaining 24.2% of the genetic variation in the dataset. The second PC accounted for 15.14% of the variation, whereas the third PC accounted for 2.06% (Figure 2b). These results show a well-structured genetic landscape among the breeds, addressing potential false-positive signals from the stratification. The data were adjusted accordingly to assess evidence for population stratification and were used as covariates for the GWAS analysis. Therefore, we retained a minimum of the first two PCs as covariates for GWAS.

**Figure 3:**
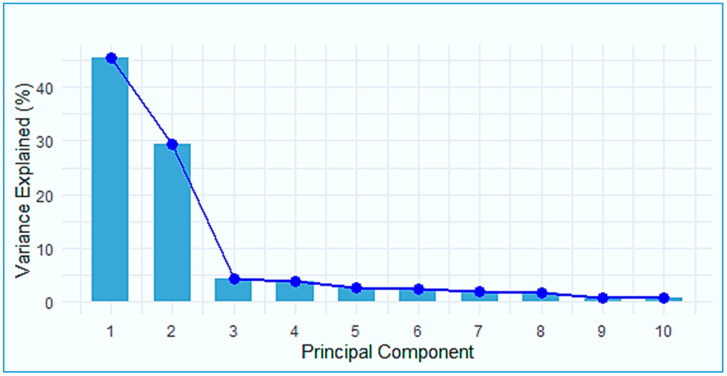
Scree plot illustrating the percentage of variance explained by each component in the three Korean commercial pig breeds based on SNP data.

The scree plot presented in Figure 3 indicates the percentage of variance explained by each principal component (PC) in the three Korean commercial pig breeds based on SNP data. This enabled us to determine the most important number of principal components that should be retained for consideration as covariates, using the elbow turning point of the bar plot indicated by the blue line, which turns at PC3. Consequently, the first two principal components (PC1 and PC2) were retained as covariates in the genome-wide association studies (GWAS).

### 3.3. The Significant SNPs and Associated Genes from GWAS

The result of the GWAS analysis for NBA and TNB in three genetically distinct background breeds is presented in Table 2, Table 3, Table 4 and Table 5. A GWAS was done on the NBA and TNB traits in the Duroc, Landrace, and Yorkshire breeds. The results show the important SNPs that were found, along with their locations, *p*-values, alleles, β values, distances, and the candidate genes that are linked to them. Each SNP was associated with a potential gene on a particular chromosome at a certain base pair (bp) location. The adjusted *p*-value showed how statistically significant the link was between a certain SNP and a trait that was being studied. Alleles denote different variants at each SNP locus, whereas the β value indicates the effect size of the SNP on the trait. The values presented in the distance column indicate the genomic distance between the SNP and the candidate genes. 

A GWA study conducted on the Duroc breed genome identified potential SNPs associated with NBA and TNB, as presented in Table 2. Two SNPs of particular interest are located on *Sus Scrofa* chromosomes (SSC) 13 and 8. The SNP on SSC 13 (*Affx-115053386*) showed an association with TNB and NBA, with suggestive threshold *p*-values of 1.52 × 10^−6^ and genome-wide threshold *p*-values of 9.56 × 10^−7^, respectively, and it was located within an unknown gene named *ENSSSCG00000063239*. Similarly, the SNP on SSC 8 (*WU_10.2_8_18342200*) was associated with both traits, with a suggestive threshold line with *p*-values of 2.01 × 10^−6^ for TNB and within a genome-wide threshold line of *p*-value 8.68 × 10^−7^ for NBA; however, there was an unknown gene named *ENSSSCG00000056996*. Additionally, SNPs associated with NBA were detected on SSC 9 and 17, with *SIDT2* and *TGM2* identified as potential candidate genes, respectively.

In Landrace breeds, SNPs on SSCs 1, 9, 3, and 14 show significant associations with TNB and NBA as presented in Table 3. The SNP on SSC 9 (*ALGA0053627*) was associated with the *PPP1R9A* gene and found within the suggestive threshold *p*-value of 2.33 × 10^−6^ for TNB and 1.55 × 10^−6^ for NBA. The SNP on SSC 3 (ALGA0017853) was associated with *LMTK2*, and the SNP on SSC 14 (*ALGA0076573*) was associated with *GTF2H3*. Across the three chromosomes of *Sus scrofa* (SSCs 9, 3, and 14), three candidate genes were associated with both TNB and NBA. These genes are *Protein Phosphatase 1 Regulatory Subunit 9A* (*PPP1R9A*), *Lemur Tyrosine Kinase 2* (*LMTK2*), and *General Transcription Factor IIH Subunit 3* (*GTF2H3*).

In the Yorkshire breed genome, GWAS identified several potential candidate genes associated with NBA (Table 4) and TNB (Table 5). These include *GRID1* (*Glutamate Ionotropic Receptor Delta Type Subunit 1*), *DLGAP2* (*Disc Large Associated Protein 2*), and *ZZEF1* (*Zinc Finger ZZ-Type and EF-Hand Domain Containing 1*). Although candidate genes such as *Poly (ADP-Ribose) Glycohydrolase* (*PARG*), *Ring Finger Protein 17* (*RNF17*), and *NADH Oxidoreductase Complex Assembly Factor 5* (*NDUFAF5*) were identified by the GWAS on specific autosomal SSCs, they had strong associations with TNB. However, the identified candidate genes were found at different *p*-values (suggestive and genome-wide significant thresholds) and on different autosomal SSCs. Notable SNPs included were *DRGA0014176* on SSC 14 within the *GRID1* gene and *WU_10.2_15_32958696* on SSC 15 within the *DLGAP2* gene, with *p*-values of 6.43 × 10^−7^ and 6.68 × 10^−7^, respectively, which were potential candidate genes found within genome-wide significance thresholds for NBA. Additionally, the SNP *H3GA0034587* on SSC 12 within the *ZZEF1* gene also shows a significant association with NBA. On the other hand, SNPs like *ASGA0064844* on SSC 14 within the *PARG* gene and *MARC0024643* on SSC 11 within the *RNF17* gene, with *p*-values of 6.20 × 10^−7^ and 7.57 × 10^−7^, respectively, indicated potential candidate genes associated with TNB found within genome-wide significant thresholds. Another SNP, *H3GA0048059* on SSC 17 near the *NDUFAF5* gene, with a *p*-value of 2.47 × 10^−6^, was also identified as another candidate gene associated with TNB found within suggestive thresholds.

### 3.4. Manhattan and Quantile–Quantile Plot

**Figure 4:**
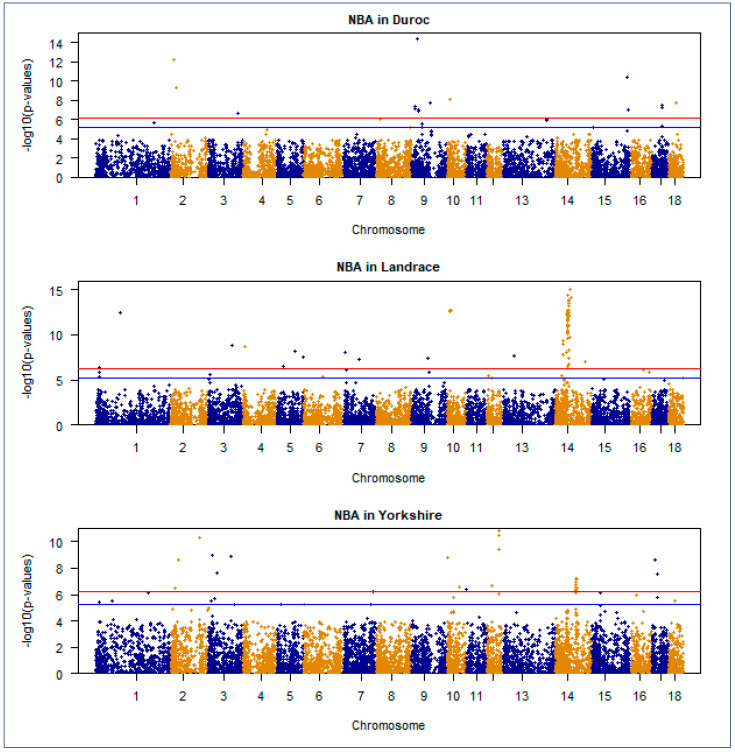
A Manhattan plot of genome−wide association studies for NBA traits in Duroc, Landrace, and Yorkshire commercial pig populations. The negative log10 *p*-values of the quantified SNPs are plotted against their genomic positions on the vertical axis. Different colors indicate different chromosomes. The horizontal red and blue lines represent genome−wide significance and chromosome−wide (suggestive) Bonferroni−corrected thresholds, respectively.

**Figure 5:**
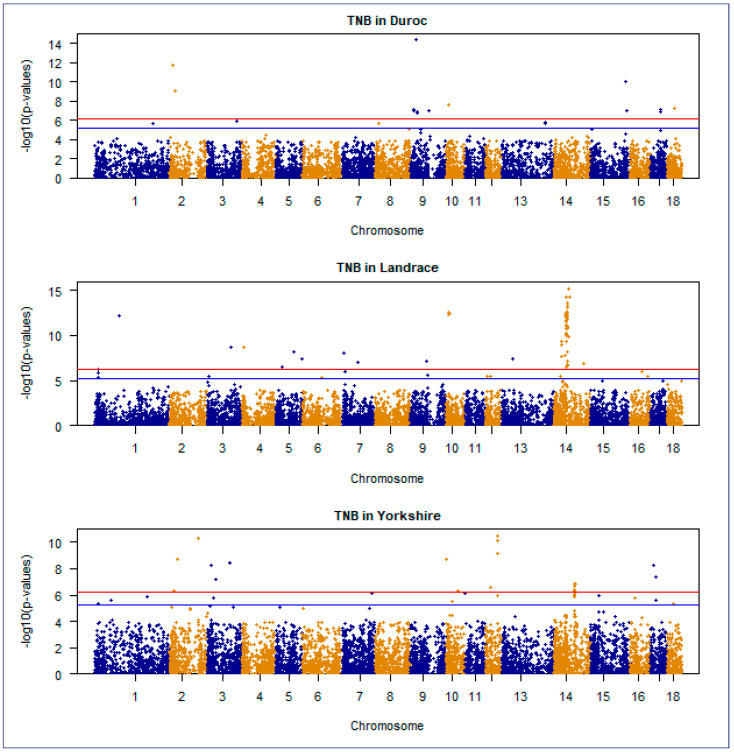
A Manhattan plot of genome−wide association studies for TNB traits in Duroc, Landrace, and Yorkshire commercial pig populations. The negative log10 *p*-values of the quantified SNPs were plotted against their genomic positions on the vertical axis. Different colors indicate various chromosomes. The horizontal red and blue lines represent genome−wide significant and chromosome−wide (suggestive) Bonferroni−corrected thresholds, respectively.

The Manhattan plot is a crucial visualization tool in GWAS that uses the inverse logarithm of *p*-values to identify statistically significant genetic variants linked to traits or diseases. A Manhattan plot of GWAS for NBA and TNB traits in Duroc, Landrace, and Yorkshire Korean breed populations were presented in Figure 4 and Figure 5, respectively.

**Figure 6:**
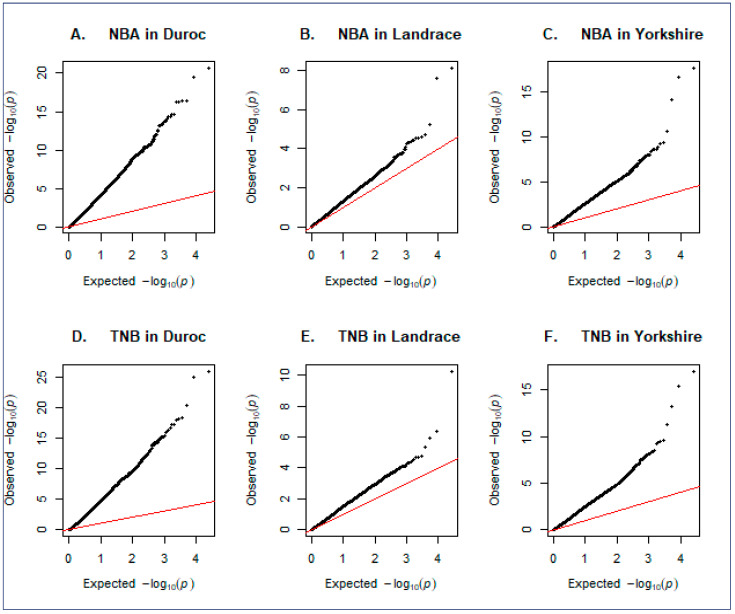
Quantile–quantile (Q–Q) plot of genome-wide association studies for NBA and TNB in Duroc, Landrace, and Yorkshire Korean commercial breeds. The Q-Q plots show the observed versus expected log *p*-values of the traits.

On the other hand, the quantile–quantile (Q–Q) plot evaluated the observed associations between genotypic SNPs and the trait under study in contrast to the null hypothesis of no association. In our study, Figure 6A–F presents the Q–Q plot of GWAS results for NBA and TNB in the Duroc, Landrace, and Yorkshire Korean breeds. For the TNB trait, the Q–Q plot for the Duroc breed (Figure 6A) demonstrated significant deviations from the expected *p*-values, indicating the presence of several SNPs with strong statistical significance. This suggests true genetic associations for the NBA trait in the Duroc breed. Similarly, the Landrace breed (Figure 6B) showed deviations from the expected line, albeit to a lesser extent than the Duroc, indicating significant but fewer associations. The Yorkshire breed (Figure 6C) also showed notable deviations, suggesting strong genetic associations, similar to the Duroc. For the TNB trait, the Q–Q plot for the Duroc breed (Figure 6D) showed substantial deviations from the expected line, indicating many significant SNPs and strong genetic associations. The Landrace breed (Figure 6E) showed a moderate number of deviations, indicating significant associations but fewer extreme *p*-values compared to the Duroc, whereas the Yorkshire breed (Figure 6F) showed deviations, indicating significant associations similar to those observed for NBA.

### 3.5. GO Annotation of Candidate Genes 

The result of GO annotations showed that *GRID1* was involved in synaptic transmission and plasticity in the central nervous system, whereas *DLGAP2* was associated with the organization of postsynaptic density and synaptic function. *ZZEF1* mainly plays a role in cell proliferation and cytoskeletal organization. *PARG* were mainly involved in DNA repair and genomic stability; *RNF17* were mostly associated with protein ubiquitination and degradation; and *NDUFAF5* participates in the assembly of mitochondrial complex I, which is crucial for cellular energy production.

### 3.6. Genes and Their Function Within Run of Homozygosity Islands

Breed-specific genes and their functions within the ROH island regions identified in our study for the Duroc, Landrace, and Yorkshire breeds are presented in Table 6. These genes reside on chromosomes 1, 12, 13, 14, and 15 of the *Sus Scrofa* chromosomes (SSC) and exhibit diverse biological functions. The genomic distribution of ROH segments revealed significant differences in the number and length of ROH segments among breeds. In the Duroc breed, we identified several genes within the ROH islands, including *NT5DC1*, *CRTAC1*, *CFAP43*, *CASC3*, *ERC2*, and *GOLGA7B*. *NT5DC1*, which is involved in nucleotide metabolism, and *CRTAC1*, which is associated with cartilage structure, occupy chromosomes 1 and 14, respectively. Additionally, we identified *CFAP43*, a gene related to cilia and flagella structure, and *CASC3*, a subunit of the exon junction complex. *ERC2*, which contributes to neuronal transmission, and *GOLGA7B*, a member of the golgin family involved in Golgi structure, have been identified on chromosomes 13 and 14, respectively. In the Landrace breed, we located the *FOCAD* gene, known as Focadhesin, within the ROH islands on chromosome 1, which is implicated in cell adhesion. Similarly, in the Yorkshire breed, we discovered *PDE6D*, encoding phosphodiesterase 6D, within the ROH islands on chromosome 15, and this enzyme participates in signal transduction processes within photoreceptor cells.

## 4. Discussion

GWAS is a powerful method to identify the mutations or genes underlying complex traits in domestic animals [39]. In the pig, GWAS has been performed to identify candidate genes for traits related to production [40], body composition [41], reproduction [1], and immunity [42]. In the present study, GWAS was conducted on the genomes of the Duroc, Landrace, and Yorkshire breeds and has identified potential SNPs associated with NBA and TNB. Population stratification is widely recognized to have an impact on the accuracy of GWAS [43]. However, unlike humans, domestic animals have a straightforward genetic makeup that facilitates the identification of specific genetic regions responsible for certain traits [39]. 

In the Duroc breed, SNPs associated with NBA were detected on SSC 9 and 17, with *SIDT2* and *TGM2* identified as candidate genes, respectively. *SIDT2* is a key protein in the autophagy-lysosomal degradation pathway and is essential for maintaining the kidney structure and function of mice [44]. However, specific research elucidating its direct involvement in pig reproductive traits (NBA) is limited. *TGM2* is known for its role in cell adhesion, migration, and differentiation, particularly in bone marrow mesenchymal stem cells (BMSCs), where it activates Wnt/β-catenin signaling, promoting cell migration and differentiation [45]. However, *TGM2*’s involvement in tumorigenesis and inflammatory processes suggests that it may also affect reproductive traits by influencing the uterine environment and embryonic development [46]. Moreover, *TGM2* encodes transglutaminase 2, an enzyme involved in protein crosslinking, apoptosis, and the pathogenesis of celiac disease [47]. It is also implicated in the inflammation-induced progression of breast cancer and may play a role in epithelial-to-mesenchymal transition (*EMT*) and cancer stem cell traits in women [48]. 

For Landrace breeds, the candidate genes identified were involved in a range of cellular processes, including synaptic function, cell proliferation, and DNA repair. *PPP1R9A* is involved in regulating protein phosphatase 1 activity, affecting synaptic plasticity and dendritic spine morphogenesis, and its role in synaptic plasticity could influence the neuroendocrine control of reproduction [47,48]. Recent evidence suggests that *LMTK2* is involved in neurodegeneration [49]. *GTF2H3* is a component of the transcription factor IIH complex, and it is crucial for transcription initiation and DNA repair. Whole-exome sequencing identified a single nucleotide variant (c.664T>C) in the *GTF2H3* gene, which appears to be the probable cause of infertility in a Turkish family [50].

In Yorkshire breeds, candidate genes associated with both NBA and TNB have been implicated in synaptic function, cell proliferation, and energy production. The roles of *GRID1* and *DLGAP2* in synaptic transmission may influence neuroendocrine regulation of reproductive processes. *GRID1* may affect the onset of puberty in female rats by regulating the levels of GnRH and RFRP-3 in the hypothalamus, as well as the concentrations of P4, but it does not affect reproductive performance in female rats [51]. Additionally, *ZZEF1*’s involvement in cell proliferation is crucial for embryonic development. However, Yu, Tencer [52] suggested that *ZZEF1* binds to the histone H3 tail and promotes KLF9/6-mediated gene regulation. Furthermore, *PARG*, *RNF17*, and *NDUFAF5* are linked to DNA repair, protein degradation, and energy production, respectively, which are vital to maintaining cellular integrity and supporting the high-energy demands of pregnancy. However, *RNF17* blocks the promiscuous activity of PIWI proteins in the mouse testes [53]. *RNF17*, a regulator of adult meiotic piRNA content, intricately modulates Piwi-interacting RNAs (piRNAs), which are crucial for gene expression regulation, especially in germline cells, by suppressing the production of secondary piRNAs [53]. 

In our previous studies [54], we delineated the genomic distribution of the run of homozygosity across three Korean breeds. In the present study, we identified and discussed the gene function in the regions of homozygosity (ROH) islands within the genomes of three pig breeds. Eight genes were identified within the ROH island, six of which are specific to the Duroc, one to the Landrace, and one to the Yorkshire breed, each of which has a specific function and is crucial for diverse biological processes and genetic disorders. Among these, *NT5DC1*, in the Duroc breed, was found to be linked to metaphyseal chondrodysplasia, highlighting its role in skeletal development. Similarly, we identified *CFAP43*, situated within another ROH island, which has been implicated in spermatogenic failure 19, emphasizing its importance in male fertility. *CFAP43* is involved in the regulation of the beating frequency of tracheal cilia, and the loss of *CFAP43* causes severe mucus accumulation in the nasal cavity in mouse and Xenopus [55]. Likewise, morphant and crispant frog embryos revealed impaired function of motile cilia in the larval epidermis, a model for airway mucociliary epithelia [55]. *CFAP43* participates in the formation of flagellar axonemes during spermatogenesis as mice mutant for Cfap43 display male infertility consistent with observations in male sterile patients [55]. Mutations in *CFAP43* lead to severe asthenozoospermia and multiple morphological abnormalities of the sperm flagellum (MMAF) in both humans and mice [56]. Moreover, we identified genes such as *CRTAC1, CASC3*, and *ERC2* within the ROH islands, indicating their involvement in ion binding, intracellular mRNA localization, and cellular component organization, respectively, in the Duroc breed. However, overexpression of *CRTAC1* increased the sensitivity to cisplatin in vitro, whereas *CRTAC1* knockdown decreased the chemosensitivity of *NSCLC* cells [57]. In vivo mouse experiments showed that *CRTAC1* overexpression increased the anti-tumor effects of cisplatin [57]. *CASC3* forms cytoplasmic condensates, and deletion of the conserved SELOR domain reduces condensate size 7-fold and significantly decreases antiviral activity towards TCV [58]. *CASC3* is a peripheral EJC protein that tailors the transcriptome by promoting degradation of EJC-dependent NMD substrates [59]. Moreover, *GOLGA7B*, *FOCAD*, and *PDE6D*, identified in ROH islands, are associated with golgin A7 family member B, focal adhesion processes, and GTPase inhibitor activity, respectively, shedding light on their roles in cellular transport, adhesion, and signaling regulation. Palmitoylation of Golga7b prevents clathrin-mediated endocytosis of DHHC5 and stabilizes it in the plasma membrane [60]. It is critical to acknowledge that further biological experiments in large swine populations will improve the reliability and understanding of candidate genes’ association with NBA and TNB traits.

## 5. Conclusions

GWAS conducted on the genomes of three Korean commercial pig breeds identified with significant SNPs associated with NBA and TNB traits, each linked to specific candidate genes on chromosomes at defined positions. Breed-specific candidate genes were identified with the NBA and TNB traits of each breed. In the Duroc breed, SNPs associated with NBA were found on SSC 9 and 17, with *SIDT2* and *TGM2* as candidate genes, respectively. In the Landrace breed, *PPP1R9A*, *LMTK2*, and *GTF2H3* have been identified across SSCs 9, 3, and 14. Similarly, candidate genes were identified in the Yorkshire breed genome for both NBA and TNB traits. Furthermore, six genes (*NT5DC1*, *CRTAC1*, *CFAP43*, *CASC3*, *ERC2*, and *GOLGA7B*) within the ROH islands of the Duroc breed were associated with metaphyseal chondrodysplasia, ion binding, spermatogenic failure, intracellular mRNA localization, and cellular component organization. Additionally, *FOCAD* was associated with Focadhesin in the Landrace breed, whereas *PDE6D* to GTPase inhibitor activity was found in the Yorkshire breed. These findings offer insights into the genetic basis of reproductive traits (NBA and TNB) in pig breeds, thereby aiding future breeding strategies to enhance sow productivity and herd performance.

## Figures and Tables

**Table 1:** Descriptive statistics of NBA and TNB in three Korean breeds.

Traits	Duroc		Landrace		Yorkshire	
	*n*	Mean ± SD	*n*	Mean ± SD	*n*	Mean ± SD
NBA	1162	7.60 ± 2.65	1782	12.42 ± 3.11	2363	13.06 ± 3.27
TNB	1162	9.26 ± 2.92	1783	13.61 ± 3.20	2364	14.61 ± 3.52

**Table 2:** Potential SNPs and candidate genes identified by the genome-wide association study of NBA and TNB in Duroc breeds. None of the displayed SNPs exceeded the genome-wide threshold or were unmarked, indicating that they met the suggestive threshold.

Trait	SSC	SNP	Position	*p*-Value	Allele	β	Distance	Gene	Gene ID
TNB	13	Affx-115053386	181,341,264	1.52 × 10^−6^	G/A	12.11	within	*Unknown*	ENSSSCG00000063239
TNB	8	WU_10.2_8_18342200	18,342,200	2.01 × 10^−6^	A/C	−10.84	within	*Unknown*	ENSSSCG00000056996
NBA	8	WU_10.2_8_18342200	18,342,200	8.68 × 10^−7^	A/C	−6.498	within	*Unknown*	ENSSSCG00000056996
NBA	13	Affx-115053386	181,341,264	9.56 × 10^−7^	G/A	6.483	131	*Unknown*	ENSSSCG00000063239
NBA	9	DRGA0009521	44,552,360	3.23 × 10^−6^	G/A	−6.292	within	*SIDT2*	ENSSSCG00000015074
NBA	17	ASGA0076732	41,226,448	5.07 × 10^−6^	G/A	6.22	4811	*TGM2*	ENSSSCG00000023522

TNB: total number born; NBA—number born alive; SSC—*Sus Scrofa* chromosomes, SNP—single-nucleotide polymorphisms; ’Distance’ columns are base pairs; ‘within’ means within the gene given in the next column on the right side.

**Table 3:** Potential SNPs and candidate genes identified by the genome-wide association study of NBA and TNB in Landrace breeds. None of the displayed SNPs exceeded the genome-wide threshold or were unmarked, indicating that they met the suggestive threshold.

Traits	SSC	SNP	Position	*p*-Value	Allele	β	Distance	Genes	Gene ID
TNB	1	WU_10.2_1_13788588	13,788,588	1.35 × 10^−6^	G/A	8.778	within	*Unknown*	ENSSSCG00000004081
TNB	9	ALGA0053627	74,677,600	2.33 × 10^−6^	A/G	−8.525	within	*PPP1R9A*	ENSSSCG00000015329
TNB	3	ALGA0017853	5,346,840	3.32 × 10^−6^	A/G	−12.24	within	*LMTK2*	ENSSSCG00000007599
TNB	14	ALGA0076573	29,354,768	3.38 × 10^−6^	A/C	−8.139	within	*GTF2H3*	ENSSSCG00000009769
NBA	1	WU_10.2_1_13788588	13,788,588	1.34 × 10^−6^	G/A	8.65	within	*Unknown*	ENSSSCG00000004081
NBA	9	ALGA0053627	74,677,600	1.55 × 10^−6^	A/G	−8.483	within	*PPP1R9A*	ENSSSCG00000015329
NBA	3	ALGA0017853	5,346,840	2.37 × 10^−6^	A/G	−12.17	within	*LMTK2*	ENSSSCG00000007599
NBA	14	ALGA0076573	29,354,768	3.06 × 10^−6^	A/C	−8.039	within	*GTF2H3*	ENSSSCG00000009769

TNB—total number born; NBA—number born alive; SSC—*Sus Scrofa* chromosomes, SNP—single-nucleotide polymorphisms; ‘Distance’ columns are base pairs; ‘within’ means within the gene given in the next column on the right side.

**Table 4:** Potential SNPs and candidate genes identified by the genome-wide association study of NBA in Yorkshire breeds. SNPs that surpass the genome-wide threshold are displayed in black and bold, whereas the remaining unmarked SNPs meet the suggestive threshold.

Trait	SSC	SNP	Position	*p*-Value	Allele	β	Distance	Genes	Gene ID
NBA	14	**DRGA0014176**	86,922,816	**6.43 × 10** **^−7^**	A/G	9.944	within	***GRID1***	**ENSSSCG00000010352**
NBA	15	**WU_10.2_15_32958696**	32,958,696	**6.68 × 10** **^−7^**	G/A	8.485	within	***DLGAP2***	**ENSSSCG00000015746**
NBA	14	**DRGA0014177**	86,969,008	**6.68 × 10** **^−7^**	A/C	9.935	within	***GRID1***	**ENSSSCG00000010352**
NBA	1	**MARC0036130**	218,896,192	**7.39 × 10** **^−7^**	A/G	−15.73	within	*Unknown*	**ENSSSCG00000039823**
NBA	12	**H3GA0034587**	50,146,896	**8.41 × 10** **^−7^**	G/A	−18.33	within	***ZZEF1***	**ENSSSCG00000032049**
NBA	10	WU_10.2_10_23632408	23,632,408	1.58 × 10^−6^	G/A	−9.205	within	*Unknown*	ENSSSCG00000036948
NBA	17	H3GA0048059	22,512,720	1.80 × 10^−6^	A/G	−9.605	3838	*SEL1L2*	ENSSSCG00000007078
NBA	1	ASGA0003159	67,590,048	2.86 × 10^−6^	G/A	−8.503	within	*ASCC3*	ENSSSCG00000004358
NBA	18	WU_10.2_18_26226731	26,226,732	3.21 × 10^−6^	A/G	−12.03	within	*KCND2*	ENSSSCG00000016621
NBA	3	ASGA0014253	11,933,535	3.36 × 10^−6^	A/G	9.992	within	*RCC1L*	ENSSSCG00000007723
NBA	1	WU_10.2_1_14946807	14,946,807	3.56 × 10^−6^	A/G	−9.535	within	*AKAP12*	ENSSSCG00000031733
NBA	3	ASGA0097775	107,125,992	5.56 × 10^−6^	A/G	−8.23	within	*BIRC6*	ENSSSCG00000008513
NBA	5	ASGA0024707	15,346,437	5.66 × 10^−6^	A/G	8.811	1196	*SPATS2*	ENSSSCG00000000199

NBA—number born alive; SSC—*Sus Scrofa* chromosomes, SNP—single-nucleotide polymorphisms; ‘Distance’ columns are base pairs; ‘within’ means within the gene given in the next column on the right side.

**Table 5:** Potential SNPs and candidate genes identified by the genome-wide association study of TNB in Yorkshire breeds. SNPs that surpass the genome-wide threshold are displayed in black and bold, whereas the remaining unmarked SNPs meet the suggestive threshold.

Trait	SSC	SNP	Position	p-Value	Allele	β	Distance	Genes	Gene ID
TNB	14	**ASGA0064844**	90,362,768	**6.20** **× 10** **^−^** **^7^**	A/C	10.05	within	***PARG***	**ENSSSCG00000010396**
TNB	11	**MARC0024643**	173,494	**7.57** **× 10** **^−^** **^7^**	A/G	−19.93	within	***RNF17***	**ENSSSCG00000009572**
TNB	14	**MARC0088303**	86,898,064	**9.56** **× 10** **^−^** **^7^**	A/G	10.01	within	***GRID1***	**ENSSSCG00000010352**
TNB	15	WU_10.2_15_32958696	32,958,696	1.03 × 10^−-6^	G/A	8.489	within	*DLGAP2*	ENSSSCG00000015746
TNB	12	H3GA0034587	50,146,896	1.11 × 10^−6^	G/A	−18.4	within	*ZZEF1*	ENSSSCG00000032049
TNB	14	DRGA0014176	86,922,816	1.22 × 10^−6^	A/G	9.901	within	*GRID1*	ENSSSCG00000010352
TNB	14	DRGA0014177	86,969,008	1.27 × 10^−6^	A/C	9.892	within	*GRID1*	ENSSSCG00000010352
TNB	1	MARC0036130	218,896,192	1.33 × 10^−6^	A/G	−15.68	within	*Unknown*	ENSSSCG00000039823
TNB	17	H3GA0048059	22,512,720	2.47 × 10^−6^	A/G	−9.632	3939	*NDUFAF5*	ENSSSCG00000007076
TNB	1	ASGA0003159	67,590,048	2.67 × 10^−6^	G/A	−8.608	within	*ASCC3*	ENSSSCG00000004358
TNB	10	WU_10.2_10_23632408	23,632,408	2.77 × 10^−6^	G/A	−9.178	within	*Unknown*	ENSSSCG00000036948
TNB	18	WU_10.2_18_26226731	26,226,732	4.17 × 10^−6^	A/G	−12.07	within	*KCND2*	ENSSSCG00000016621
TNB	1	WU_10.2_1_14946807	14,946,807	5.08 × 10^−6^	A/G	−9.55	within	*AKAP12*	ENSSSCG00000031733

NBA—number born alive; SSC—*Sus Scrofa* chromosomes, SNP—single-nucleotide polymorphisms; ‘Distance’ columns are base pairs; ‘within’ means within the gene given in the next column on the right side.

**Table 6:** Breed specific genes and their function within ROH island regions in three breeds.

Breed	Gene ID	Genes	SSC	Start (bp)	End (bp)	Pos (Mbp)	Function
DD	ENSSSCG00000004435	*NT5DC1*	1	81,746,022	81,881,113	81.75	Metaphyseal Chondrodysplasia
DD	ENSSSCG00000010530	*CRTAC1*	14	109,223,293	109,390,691	109.22	Ion binding
DD	ENSSSCG00000010609	*CFAP43*	14	115,073,366	115,174,043	115.07	Spermatogenic failure 19
DD	ENSSSCG00000017477	*CASC3*	12	22,199,228	22,221,499	22.20	Intracellular mRNA localization
DD	ENSSSCG00000024417	*ERC2*	13	37,388,517	38,367,504	37.39	Cellular component organization
DD	ENSSSCG00000040359	*GOLGA7B*	14	109,210,866	109,225,153	109.21	Golgin A7 family member B
LL	ENSSSCG00000028406	*FOCAD*	1	201,942,463	202,258,794	201.88	Focadhesin
YY	ENSSSCG00000016279	*PDE6D*	15	132,375,743	132,426,683	132.38	GTPase inhibitor activity

DD—Duroc; LL—Landrace; YY—Yorkshire; SSC—*Sus Scrofa* chromosomes, ROH—run of homozygosity; Pos—position.

## Data Availability

The data presented in this study are available on request from the corresponding author due to the privacy of the breeding farm.
